# Acid Stress Response Mechanisms of Group B Streptococci

**DOI:** 10.3389/fcimb.2017.00395

**Published:** 2017-09-07

**Authors:** Sarah Shabayek, Barbara Spellerberg

**Affiliations:** ^1^Institute of Medical Microbiology and Hygiene, University of Ulm Ulm, Germany; ^2^Department of Microbiology and Immunology, Faculty of Pharmacy, Suez Canal University Ismailia, Egypt

**Keywords:** *Streptococcus agalactiae*, acid resistance, low pH, molecular mechanisms, stress response

## Abstract

Group B streptococcus (GBS) is a leading cause of neonatal mortality and morbidity in the United States and Europe. It is part of the vaginal microbiota in up to 30% of pregnant women and can be passed on to the newborn through perinatal transmission. GBS has the ability to survive in multiple different host niches. The pathophysiology of this bacterium reveals an outstanding ability to withstand varying pH fluctuations of the surrounding environments inside the human host. GBS host pathogen interations include colonization of the acidic vaginal mucosa, invasion of the neutral human blood or amniotic fluid, breaching of the blood brain barrier as well as survival within the acidic phagolysosomal compartment of macrophages. However, investigations on GBS responses to acid stress are limited. Technologies, such as whole genome sequencing, genome-wide transcription and proteome mapping facilitate large scale identification of genes and proteins. Mechanisms enabling GBS to cope with acid stress have mainly been studied through these techniques and are summarized in the current review

## Introduction

*Streptococcus agalactiae* or group B streptococcus (GBS) is an opportunistic pathogen which colonizes up to 30% of the genitourinary and gastrointestinal tracts of healthy women. At the same time GBS is a leading cause of life-threatening neonatal infections, such as meningitis, sepsis and pneumonia (Verani et al., 2010; Le Doare and Heath, 2013). A primary risk factor for GBS transmission to newborns is maternal colonization at birth, where GBS may spread either *in utero* by ascending infection or intrapartum through the aspiration of contaminated vaginal or amniotic fluids (Maisey et al., 2008a; Melin, 2011; Le Doare and Heath, 2013). Every tenth neonate may acquire GBS, which has also been proposed as a normal element of the neonatal microbiome (Landwehr-Kenzel and Henneke, 2014). Although GBS is harmless as a colonizer of healthy women, it can cause serious infections in pregnancy. In addition, GBS has been increasingly reported as being responsible for invasive disease in elderly and immunocompromised patients (Farley, 2001; Maisey et al., 2008a; Melin, 2011; Le Doare and Heath, 2013).

**Figure 1:**
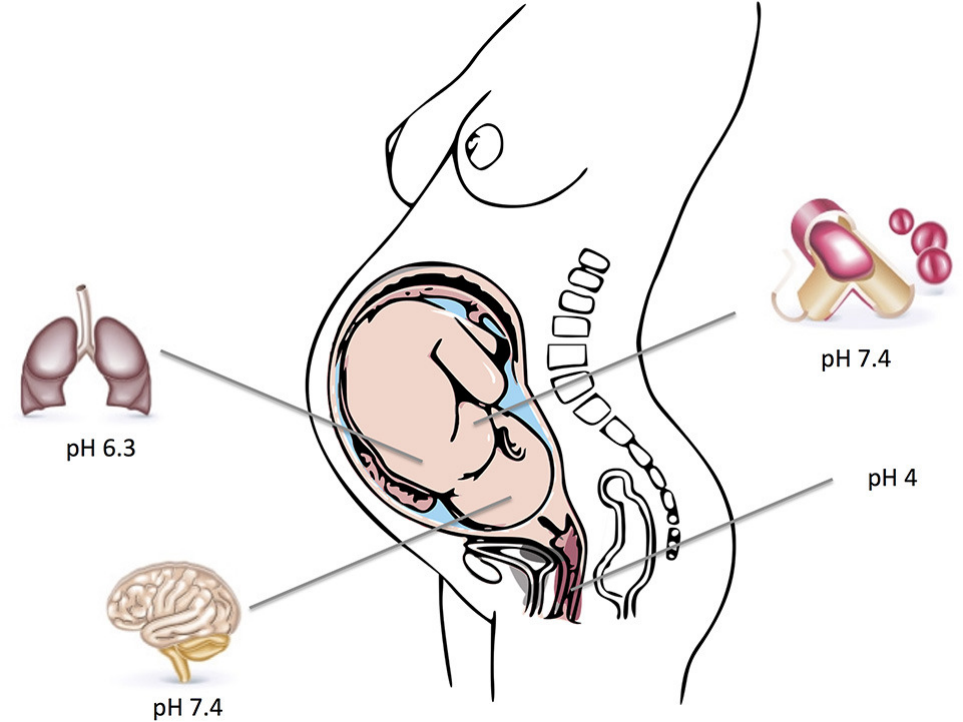
Depicted are typical host environments and their respective pH values that GBS can colonize and infect. Parts of the figure designed by Freepik.

Assessment of GBS pathophysiology reveals that it has the capability to survive in various environments within the human host. It typically colonizes the vaginal mucosa, but also causes different types of invasive infections. GBS have thus successfully adapted to varying pH levels ranging from the acidic environment of the vagina or intracellular compartments to the almost neutral pH-values of amniotic fluid, the respiratory tract and human blood. These changes are most likely achieved by modifying the transcription of pathogen-host interaction related genes. The typical host environments and their respective pH-values that GBS can colonize and infect are depicted in Figure 1.

In contrast to other Streptococci (Cotter and Hill, 2003; Kajfasz and Quivey, 2011), investigations on GBS responses to acid stress are limited. Whole GBS genomes sequencing projects (Glaser et al., 2002; Tettelin et al., 2002, 2005) lead to the identification of several genes with high similarities to streptococcal systems known to be involved in acid stress resistance. Several large scale genome-wide investigations employing technologies, such as DNA microarrays and proteomic analysis (two-dimensional polyacrylamide gel electrophoresis 2D-PAGE in combination with mass spectrometry) focused on pH-responsive GBS genes and facilitated the identification of new targets that are induced under acid stress (Cotter and Hill, 2003; Martin-Galiano et al., 2005; Bore et al., 2007; Gong et al., 2009; Martinez et al., 2010).

In general, streptococci possess an array of different defense mechanisms to cope with low pH. Proton pumps represent the most direct approach by transporting protons outside the cell to keep a proper level of intracellular pH. Inducing a buffering effect through increasing the concentration of intracellular alkaline compounds is another approach used to counteract cytoplasmic acidification. Additional mechanisms include repair or prevention of acid damage in macromolecules and modifying proton permeability of the cellular membrane. Regulation and control of these mechanisms is exerted through Two-component systems (TCSs), transcriptional regulators and sigma factors which respond to acid stress by modifying gene expression. Moreover, metal ion homeostasis, osmoregulation and oxidative stress response have been increasingly reported to contribute to acid adaptation mechanisms.

**Figure 2:**
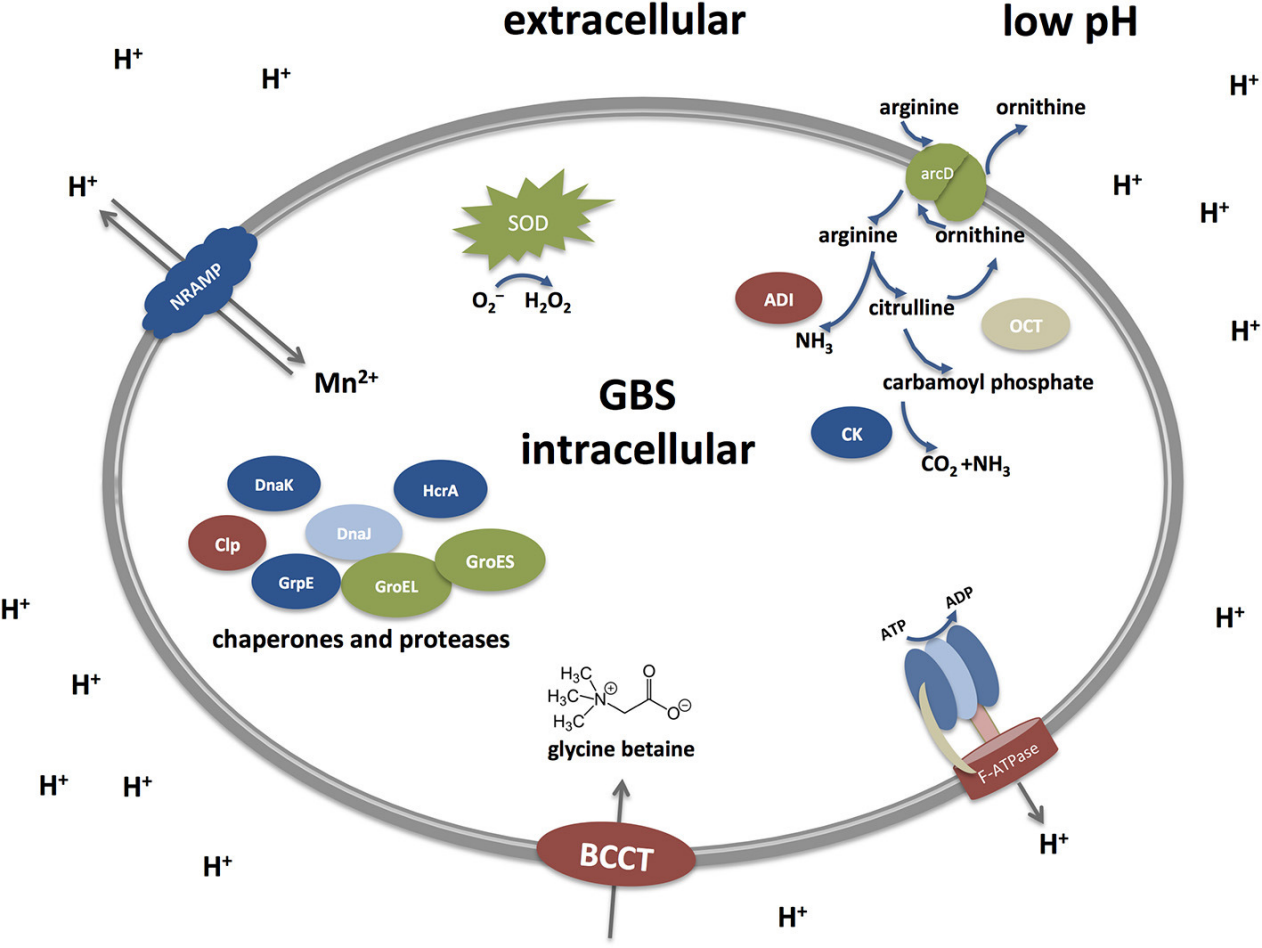
Acid stress responses in *Streptococcus agalactiae* (Group B streptococci, GBS) under low pH. GBS possess different defense mechanisms to cope with low pH. They include the Arginine deiminase system (ADI), an F-ATPase transporter, transporter of the BCCT family, chaperones and proteases, SodA, and a NRAMP-type transporter. The Arginine deiminase system (ADI) comprises three units: an arginine deiminase (AD), ornithine carbamoyltransferase (OTC) and carbamate kinase (CK). Arginine is taken up from the extracellular environment and cleaved by AD into citrulline and ammonia. Citrulline is further cleaved to yield ornithine and carbamoyl phosphate by the action of OTC. Finally, CK cleaves carbamoyl phosphate into carbon dioxide and ammonia, thereby generating an alkaline microenvironment. Proton pumps like the F-ATPase represent the most direct approach to counteract acid stress by transporting protons outside the cell to keep a proper level of intracellular pH. Under low pH, the F-ATPase system is induced to pump out protons extracellularly in order to maintain the alkalinity of the intracellular cytoplasm. Additional mechanisms include repair or prevention of acid damage in macromolecules by chaperones and proteases, such as Dnak, GroES, and CLp. Metal ion homeostasis also takes part in the acid response as the NRAMP metal ion symporter takes up Mn^2+^ and expels protons out of the cell. The osmotic stress is tightly controlled as well through the up-regulation of the glycine betaine osmoregulation system where choline and glycine betaine (a powerful osmoprotectant) are taken up by transporters of the BCCT (Betaine/Carnitine/Choline Transporter) family. Defense mechanisms to avoid the damaging effects of superoxide species generated during acid stress are mainly exerted through the activity of the streptococcal superoxide dismutase (SOD).

Low pH environments are often encountered by GBS inside the human host and mechanisms that enable GBS to cope with acid stress must therefore be essential for colonization as well as infection. However, our understanding of the acid stress response in GBS is incomplete. While the relevant mechanisms have often been studied at a functional level in other streptococci or gram positive pathogens studies in GBS largely rely on genomic and transcriptomic investigations. With this review, we try to summarize our current knowledge about mechanism permitting GBS survival at low pH and draw comparisons to other gram positive bacteria, especially streptococci. Figure 2 represents a simplified graph explaining different acid stress responses in GBS which will be discussed below.

## Low pH environments encountered within the human host

### The human vagina

A low pH of 4 ± 0.5 is crucial to maintain a healthy vaginal environment. These pH-values mediate a microbicidal impact, which has been demonstrated to be effective against various microbial pathogens of sexually transmitted diseases (STD), including HIV (Aldunate et al., 2013). Vaginal acidity develops during puberty due to elevated estrogen levels which promote the accumulation of glycogen in epithelial cells. The latter is then converted into acetic and lactic acids by fermentation through epithelial cells and lactobacilli of the vaginal microbiota (Boskey et al., 2001).

Lactobacilli are the dominant microbiota in the vagina of healthy women. They are responsible for maintaining vaginal acidity (Boskey et al., 1999, 2001) which inhibits colonization by bacterial pathogens and restores the vaginal ecosystem (Juarez Tomas et al., 2003). Any lactobacilli decline or eradication events render the host more prone to bacterial vaginosis (BV), genital tract infections (GTIs) by *Neisseria gonorrhoeae* or *Trichomonas vaginalis*, vulvovaginal candidiasis (VVC), and urinary tract infections (UTIs) (Falagas et al., 2007; Ruiz et al., 2009). Key findings in BV, for instance, are the overgrowth of anaerobic bacteria, diminished lactobacilli levels and most importantly an elevated vaginal pH above 4.5 (Brabin et al., 2005; Falagas et al., 2007). Lactobacilli have even been proposed as an alternative strategy for controlling GBS infections (Acikgoz et al., 2005; Ruiz et al., 2012). In addition to vaginal acidification, lactobacilli can block pathogens of the genitourinary tract by mechanisms, such as releasing adhesion-inhibiting bio-surfactants, antimicrobial substances and hydrogen peroxide as well as auto-aggregation, co-aggregating with other bacterial species and surface hydrophobicity (Ruiz et al., 2009). GBS need to be able to counteract or evade all these strategies in order to successfully colonize the vaginal mucosa.

### The phagosome

Phagocytic cells as part of the innate immune system are recruited to sites of infections in order to eliminate invading pathogens. Once recognized, microbial pathogens are phagocytosed, and incorporated into a phagosome. Phagosomal maturation then occurs leading to the creation of phagolysosomes (Flannagan et al., 2009; Russell et al., 2009).

Maturation involves alteration of the phagosomal membrane which results in an extremely acidic, oxidative and degradative environment. Early phagosome possesses a moderately acidic (pH 6.1–6.5) intraluminal pH. As maturation proceeds to later stages, the lumen becomes more acidic (pH 5.5–6.0) and enriched in proteases and lysosomal-associated membrane proteins (lAMPs). Terminal phagolysosomal compartments are strictly acidic, possessing intraluminal pH-values as low as 4.5, and contain reactive oxygen species (ROS), reactive nitrogen species (RNS), and antimicrobial peptides. Thus, during the maturation process, phagosomes become fully armed with destructive antimicrobial features (Ohkuma and Poole, 1978; Yates et al., 2007; Flannagan et al., 2009).

Despite considering GBS mainly as an extracellular pathogen, it has the ability to persist and survive within macrophages. Prolonged GBS survival within the phagolysosome has previously been shown (Valenti-Weigand et al., 1996; Cornacchione et al., 1998; Cumley et al., 2012). Surprisingly, GBS was found to be > 10-fold less prone to hydrogen peroxide killing than catalase-producing *Staphylococcus aureus* (Wilson and Weaver, 1985). Furthermore, it was recently reported that the acidification of phagosomes is essential for prolonged intracellular survival of GBS (Cumley et al., 2012) and that the pilus protein which is required for antimicrobial peptides resistance is up-regulated under acidic conditions (Maisey et al., 2008b). In line with these observations, the two-component regulator system CovS/CovR, which mediates GBS tolerance to acid stress, has been shown to be critical for survival within the phagosome (Cumley et al., 2012).

## Effect of low pH on GBS adherence and biofilm formation

Adherence to host tissues is a fundamental step preceding GBS colonization and ensuing invasion. GBS is capable to adhere and invade various host cells, including epithelial cells of the vagina and the lung, endothelial cells and micro-vascular endothelial cells of the blood brain barrier (Maisey et al., 2008a). Following the initial attachment to certain proteins, GBS interacts with integrins of the host-cell surface and often enters into these cells. Numerous proteins have been reported as adhesins contributing to GBS binding to host cells and extracellular matrix (ECM) proteins (Nobbs et al., 2009; Park et al., 2012).

As a commensal of the female genital tract, GBS colonization preferentially takes place at low vaginal pH-values. Early reports demonstrated enhanced GBS adherence at low pH to vaginal and respiratory epithelial cells (Zawaneh et al., 1979; Tamura et al., 1994). Microarray analysis (Santi et al., 2009) detected the up-regulation of the *dpsA* gene under acidic conditions, which is essential for the attachment of GBS to eukaryotic cells, at low pH (Samen et al., 2004). Furthermore, the glutamine transport gene *glnQ* (Tamura et al., 2002) required for both adherence to fibronectin *in vitro* and for virulence *in vivo* was found to be up-regulated under decreasing pH-values (Santi et al., 2009). Comparative expression analysis between GBS strains with different clinical virulence resulted in the identification of a novel adhesin designated HvgA which is a specific signature for the hypervirulent GBS ST17 strain. HvgA promotes colonization, adhesion and host invasion. It was found to be regulated by the acid-sensing two-component regulator CovS/CovR and was reported to be crucial for GBS intestinal colonization (Tazi et al., 2010).

Low pH also promotes biofilm production in GBS (Rosini and Margarit, 2015). Bacteria in biofilms form high density populations that are more resistant to stress (Nobbs et al., 2009) and contribute to the acid resistance of Gram-positive bacteria (Cotter and Hill, 2003). GBS is able to form biofilms with a maximum biofilm formation time of 48 h (Yang et al., 2012), which is influenced by environmental pH changes. Increased biofilm formation by GBS is observed at acidic pH (Ho et al., 2013; D'Urzo et al., 2014). It is noteworthy that also elevated expression levels of pilus components are observed at acidic pH (Santi et al., 2009). Pilus proteins have previously been reported to be involved in GBS epithelial adherence and biofilm formation (Konto-Ghiorghi et al., 2009; Rinaudo et al., 2010) leading to the hypothesis that a higher pilus expression in the acidic environment of the vagina may favor colonization. D'Urzo et al. (2014) investigated 389 GBS isolates for their ability to produce biofilms. They reported clones belonging to the hypervirulent ST17 serotype III GBS lineage to be the best biofilm producers forming stronger biofilms under acidic pH. The hypervirulent ST17 harbors genes encoding pili and the HvgA adhesin (Tazi et al., 2010; D'Urzo et al., 2014; Teatero et al., 2016; Perichon et al., 2017) It is the most frequent cause for neonatal meningitis relative to other GBS clones (D'Urzo et al., 2014; Landwehr-Kenzel and Henneke, 2014; Teatero et al., 2016). Furthermore, the global virulence regulator CovS/CovR has been shown to control GBS adherence and biofilm production in response to environmental pH changes (Santi et al., 2009; Park et al., 2012). According to Santi et al. (2009), CovRS mediates the upregulation of virulence determinants and controls GBS transition from colonizing to invasive state upon translocation from the acidic vagina to neutral host environments. Consistently, Patras et al. (2013) demonstrated CovRS critical in modulating the host innate immune response by restricting virulence factors expression to promote colonization and maintain the GBS commensal phenotype in the vagina (Patras et al., 2013).

## Bacterial mechanisms to cope with the acid stress

### Production of alkali: arginine deiminase system (ADI)

One of the common pathways to counteract acid stress is the release of buffering substances. The release of alkaline molecules, such as ammonia seems to be a universal approach of acid resistance in lactic acid bacteria where arginine is a well-known ammonia precursor. A classic enzymatic pathway for ammonia release is the Arginine deiminase system (ADI), it comprises an arginine deiminase, ornithine carbamoyltransferase (also referred as ornithine transcarbamylase), and carbamate kinase. These three enzymes are encoded by the *arcA, arcB* and *arcC* genes, respectively. ADI provides two moles of ammonia and one ATP unit per each molecule of arginine. To maintain pH homeostasis, ATP can be used to expel protons across the membrane while a by-product like ornithine is secreted out of the cell in place of arginine in an energy independent manner under the action of an ornithine/arginine antiporter, encoded by *arcD* (van de Guchte et al., 2002; Cotter and Hill, 2003; Kajfasz and Quivey, 2011).

Arginine deiminase system (ADI) has been characterized in oral streptococci (Burne and Marquis, 2000; Dong et al., 2002; Griswold et al., 2004; Gruening et al., 2006), *S. pyogenes* (Hering et al., 2013), and *S. pneumoniae* (Schulz et al., 2014). Complete genome projects show that GBS possess an ADI (Glaser et al., 2002; Tettelin et al., 2002, 2005) which, although not yet characterized, appears to resemble homologs in other streptococci (Griswold et al., 2004). Transcription analysis in GBS reported genes encoding a carbamate kinase and an ornithine carbamoyltransferase to be extremely up-regulated at low pH (Santi et al., 2009). Similar findings were reported during a global transcript profiling of growth phase regulated genes in GBS. The authors indicated 55–150 fold up-regulation of ADI genes during the stationary growth phase (Sitkiewicz and Musser, 2009) which can be considered as an acid stress phase for GBS as it produces lactic acid as a side-product of carbohydrate fermentation.

However, ADI function in GBS seems not to be restricted to acid tolerance. A recent proteomic study reported the up-regulation of arginine deiminase ArcA in response to human serum (Yang et al., 2011). Transcriptome analysis revealed 5–15 fold induction of *arcA* following incubation with human blood (Mereghetti et al., 2008) and about 2–3 fold increase at stationary phase growth in amniotic fluid (Sitkiewicz et al., 2009). According to Cotter and Hill (2003) environmental pH itself is not a major factor that triggers ADI expression in streptococci. Direct involvement of ADI in acid tolerance is not mandatory. Rather than low pH, ADI expression could be also driven by intracellular arginine, lack of energy, catabolite suppression or oxygenation (van de Guchte et al., 2002).

### Proton pumps: F0F1-ATPase

The F1F0-ATPase is a multi-subunit enzyme which translocates protons at the expense of ATP or conversely synthesizes ATP using protons. It is composed of a membrane-embedded F0 complex, which consists of the three subunits a, b, and c and a cytoplasmic-bound F1 complex. F0 translocates protons as a membrane-bound proton specific channel. The F1 domain comprises five subunits α, β, γ, δ, and ε. It promotes ATP synthesis during protons movement from the extracellular compartments into the cytoplasm, or cleaves ATP when protons are expelled out of the cell. The net result is a more alkaline cytoplasm in comparison to an extrinsic acidic environment (van de Guchte et al., 2002; Cotter and Hill, 2003).

Interestingly, the genetic organization of the encoding *atp* operon in streptococci, *atp*EBFHAGDC (Smith et al., 1996; Quivey et al., 2001; Cotter and Hill, 2003), is somewhat different from that described earlier for *E. coli* (Walker et al., 1984). The F0 gene order in streptococci consists of *atpEBF*. This is dissimilar to the *atpBEF* order identified in other bacteria. If this variant genetic organization is important in regard to function is however still under investigation. Generally, the F-ATPase is the primary mechanism pumping out protons in order to keep pH homeostasis of streptococci under acidic condition. The transcriptional machinery of F-ATPase appears to be similar for many streptococcal species. Transcription analysis of the F-ATPase systems demonstrated an upregulation of the encoding genes at low pH for *S. mutans, S. suis, S. sanguinis*, and *S. pneumoniae* (Martín-Galiano et al., 2001; Kuhnert et al., 2004; Gong et al., 2009; Wei et al., 2011). Genome sequencing projects have predicted GBS to possess the F-ATPase operon similar to other streptococci (Glaser et al., 2002; Tettelin et al., 2002, 2005), but a detailed functional analysis hat not been conducted in GBS. The available large scale transcriptome analysis in GBS indicated a steady or slightly increasing transcript level of the F1-ATP synthase subunits *atp*ABEF genes over time independently of growth phase (Sitkiewicz and Musser, 2009). Likewise, a recent proteomic investigation reported the abundance of the F1-ATP synthease subunit gamma during mid-exponential growth phase of GBS (Yang et al., 2010). F-ATPase activity under acid stress in GBS may thus not only be restricted to the extrusion of protons but may also be needed for ATP synthesis during growth and maintenance. Being a lactic acid producing organism, a steady accumulation of acids is expected during GBS growth and may explain the elevated transcription levels of the F1-ATP system. Supporting this interpretation, previous investigations indicated the existence of a basal level of ATPases at alkaline pH, which was attributed to allow rapid resumption of growth upon pH decrease (Kobayashi et al., 1986; Kakinuma, 1998; Cotter and Hill, 2003).

### Acid tolerance response (ATR)

Some bacterial species display elevated survival rates after exposure to lethal acidic pH provided that they are only briefly challenged with sub-lethal acidic levels. This phenomenon is recognized as the acid tolerance response (ATR) (Cotter and Hill, 2003). However, other bacteria possess additional systems to cope with acid stress at levels that are too acidic to permit growth (pH 2.5 and below). A response which is designated acid resistance (AR) or extreme acid resistance (XAR) (Lund et al., 2014). So ATR comprises mechanisms that keep intracellular pH homoeostasis, while the XAR or AR represents an extreme acid stress response that includes mechanisms to avoid the intracellular pH from dropping to life-threating levels (Cotter and Hill, 2003; Lund et al., 2014). It is a remarkable observation that F0F1-ATPase and not ADI has been frequently reported to play a role in the induction of ATR (Cotter and Hill, 2003; Lund et al., 2014). Both ATR and AR have been defined for *E. coli* (Goodson and Rowbury, 1989), *Salmonella* Typhimurium (Foster and Hall, 1990), *Lactococcus lactis*, and Lactobacilli (Lund et al., 2014) while ATR has been described in oral streptococci (Hamilton and Buckley, 1991; Nascimento et al., 2004; Papadimitriou et al., 2007; Martinez et al., 2010) and for *S. pneumoniae* (Martin-Galiano et al., 2005).

Neither ATR nor AR has been investigated or addressed in GBS. The only available study is that done by Yang et al. (2012). They observed that short term acid exposure does not drive an ATR in GBS when inoculating acid adapted cells into pH 5 and monitoring them for long term survival. The authors were not able to detect any improvement in GBS survival. Despite these findings, there remains a possibility that GBS is able to mount an ATR under different culture conditions. Nascimento et al. (2004) was able to detect ATR in *S. sobrinus* when using bacterial cells cultivated in a continuous chemostat culture in contrast to Svensäter et al. (1997) who were unable to distinguish any ATR when employing buffered media and batch-cultivated cells of *S. sobrinus*.

### Sensing acid stress and signaling

Modulation of gene expression as a consequence of varying extracellular environmental conditions is a fundamental adaptation response that is mandatory for bacteria to replicate and survive (Cotter and Hill, 2003). Alternative sigma factors, transcriptional regulators and two-component signal transduction systems (TCSs) have been demonstrated to control the coordinated gene expression in bacteria experiencing changing environmental conditions (Cotter and Hill, 2003).

Sigma factors play an essential part in the bacterial response to low pH. Analysis of the GBS genome revealed the presence of three putative sigma factors, the major sigma factor σA, ComX and an ECF-type sigma factor (Glaser et al., 2002). The later has been reported in *S. equi* but not in *S. pneumoniae, S. mutans*, or *S. pyogenes* (Glaser et al., 2002). In addition, Glaser et al. (2002) identified numerous transcriptional regulators in GBS representing 5% of the predicted genes, several of which were upregulated at pH 5.5 (Santi et al., 2009). Similar findings were made for *S. mutans* another streptococcal species highly adapted to an acidic environment (Gong et al., 2009).

Two-component signal transduction systems (TCSs) are composed of a membrane-associated histidine kinase sensor and a cytoplasmic response regulator. They contribute to adaptation, virulence and survival through detecting environmental fluctuations and providing a proper response (Cotter and Hill, 2003). The *S. mutans* genome contains 14 TCS of which LiaRS, CiaRH, ComDE, and CovRS were found to be up-regulated upon acid adaptation (Gong et al., 2009). Besides, they were formerly reported to participate in acid stress and considered as crucial virulence elements in *S. mutans* (Li et al., 2002; Ahn et al., 2006; Lévesque et al., 2007; Gong et al., 2009; Kawada-Matsuo et al., 2009). The GBS genome was found to encode as many as 20 sensor histidine kinases and 21 response regulators (Glaser et al., 2002). Interestingly genome comparison revealed a much higher number of TCSs in GBS than those reported in related species, such as *S. pyogenes, S. pneumoniae, S. mutans*, and *L. lactis* suggesting a higher capacity of GBS to adapt to varying environmental conditions (Glaser et al., 2002). However, only few TCSs have been characterized in GBS to date (Klinzing et al., 2013). One of the most well-studied TCSs is the CovRS system (or CsrRS) (Faralla et al., 2014). It is best known as a major virulence regulator of pathogenic streptococci and its direct contribution to acid stress tolerance has previously been reported for GBS (Lamy et al., 2004; Santi et al., 2009; Firon et al., 2013; Faralla et al., 2014; Perichon et al., 2017). Genome-wide transcription analysis found 90% of the down-regulated genes and 60% of the up-regulated genes at pH 5.5 were CovRS dependent (Santi et al., 2009). Another TCS in GBS is the CiaR/H system. Consistent to *S. mutans*, reports on GBS demonstrated CiaR/H contribution to acid adaptation and enhancing intracellular survival in macrophages (Quach et al., 2009). The CiaR/H-dependent genes in GBS have shown significant homology to acid and multi-stress tolerant genes of *L. lactis* (Quach et al., 2009). Furthermore, recent microarray analysis proposed the involvement of CiaR/H in acid tolerance of *S. suis* (Wei et al., 2011).

### Osmoregulation

Since most bacteria do not possess active water transport mechanisms to maintain cell turgor, alternative approaches have been developed to endure osmotic stress (Poolman et al., 2002). Shifts in osmotic pressure result in altered gene expression patterns of transporters or enzymes to sustain water equilibrium (Poolman et al., 2002). One of the well-known bacterial strategies in response to osmotic stress is to accumulate solutes, such as glycine betaine, proline, carnitine, and choline as osmoprotectants. These are soluble zwitterionic substances which can be retrieved from the surrounding milieu or biosynthesized intracellularly in high concentrations without interfering in critical physiological pathways (Kempf and Bremer, 1998).

Evidence for a putative connection between surviving acidic conditions and osmotic stress was reported by Santi et al. for GBS (Santi et al., 2009). They found an up-regulation of genes encoding components of the glycine betaine osmoregulation system upon shifting GBS from pH 7 to pH 5.5. The glycine betaine system has previously been reported to counteract osmotic pressure in *Bacillus subtilis* (Kempf and Bremer, 1995, 1998) and *L. lactis* (Obis et al., 1999). Consistently, in *S. mutans* it was observed that acid tolerance responses following an acid shock from pH 7.5 to pH 5.5 may protect against osmotic stress (Svensater et al., 2000). A potential connection between ATR and osmotic stress is supported by findings in *S. pneumoniae* that showed an increased expression level of a choline transporter under acid stress (Martin-Galiano et al., 2005). A choline transporter is comparable to the glycine betaine transporter of *B. subtilis* (Kappes et al., 1996) where an osmotically mediated choline uptake is also reported (Kappes et al., 1999). Choline is a necessary precursor for the biosynthesis of glycine betaine (Kappes et al., 1996, 1999).

### Protection or repair of macromolecules: chaperones

Rapid adaptive bacterial responses to abrupt environmental changes involve the release of proteases and chaperones in order to protect and repair macromolecules like DNA and proteins which are essential for optimal acid adaptation (Cotter and Hill, 2003). Chaperones provide protection against different environmental stresses by aiding in protein folding, renaturation, and eradication of damaged proteins (Cotter and Hill, 2003). The most common bacterial molecular chaperones include DnaK, DnaJ, GrpE and HrcA, which are encoded by the DnaK operon (*hrcA*-*grpE*-*dnaK*-*dnaJ*), GroEL and GroES, encoded by the GroE operon (*groES*-*groEL*), and Clp proteases (Jayaraman et al., 1997; Lemos et al., 2001; Nair et al., 2003; Henderson et al., 2006; Tomoyasu et al., 2012). DnaK is an ATP-dependent chaperone that works together with the co-chaperone DnaJ and the nucleotide exchange factor GrpE. It prevents protein aggregation and ensures proper folding by binding to hydrophobic peptides sequences during protein synthesis. GroEL possesses an interior cavity where substrates are protected by the union of GroES to GroEL in an ATP-dependent manner. Both DnaK and GroEL are negatively regulated by HrcA (Lemos et al., 2001; Wong and Houry, 2004). The Clp ATPases constitutes a huge family of highly conserved and universal proteins that are present in both prokaryotic and eukaryotic organisms. They act as molecular chaperones and contribute to protein assembly, folding, as well as proteolysis (Nair et al., 2003).

Chaperones and proteases are greatly conserved throughout different genomes implying their fundamental tasks for cellular life (Jordan et al., 2002). A clear linkage between acid stress and chaperones can be demonstrated in numerous Gram-positive bacteria. *S. mutans* grown in continuous chemostate culture displayed a marked increase in DnaK levels in response to acid shock (Jayaraman et al., 1997). Supporting these findings an up-regulated expression of DnaK, DnaJ and GroEL in response to acidic stimuli were reported for *S. mutans* in different studies (Lemos et al., 2001; Wilkins et al., 2002; Matsui and Cvitkovitch, 2010). Moreover, similar results were found for *S. pneumoniae* (Martin-Galiano et al., 2005) and *S. sobrinus* (Nascimento et al., 2004) under acid stress. Consistently, proteomic investigation in GBS identified GroES among the more abundantly expressed proteins in cells grown at pH 5 (Yang et al., 2010). Sitkiewicz and Musser (2009) could demonstrate the genes *hrcA, grpE, dnaK* to be significantly up-regulated in the more acidic stationary growth phase of GBS. However, a different scenario was presented by Santi et al. (2009) as genes belonging to the *hrcA-grpE* operon were detected to be down-regulated upon shifting from pH 7 to pH 5.5. Interestingly a down-regulated DnaK operon during acid shock was also found by Wei et al. (2011) in *S. suis*. In agreement, a *S. intermedius dnaK* mutant could not show significant acid sensitivity (Tomoyasu et al., 2012). One possible explanation for the contradictory results in GBS could be attributed to differences in growth conditions and most notably the brief exposure time to acid stress (30 min) utilized by Santi et al. (2009).

The Clp chaperones have also been linked to acid tolerance responses. Previous studies in *S. mutans* employing a ClpL-deficient mutant exhibited impaired viability of the mutant under acidic conditions in comparison to the parent strain (Lemos and Burne, 2002; Kajfasz et al., 2009). Likewise, Len et al. (2004) found ClpL protein levels to increase under acidic pH in *S. mutans*. The gene encoding ClpL protease was also up-regulated in response to acid in *S. pneumoniae* (Martin-Galiano et al., 2005) and reported to take part in ATR response where an early activation of ClpL (5 min after acid shock) could be detected. Genome analysis revealed (Glaser et al., 2002), that GBS encodes a bulk group of Clp protease subunits, which include the ClpP proteolytic subunit, four ATPase regulatory subunits; ClpX, ClpC, ClpL, ClpE, and three identical ClpA ATPase paralogs. Characterization of the ClpP serine protease in GBS reported its critical involvement in growth regulation under stress conditions (Nair et al., 2003). Moreover, elevated transcriptions of *clpE* and *clpL* in GBS during the more acidic stationary phase have been observed (Sitkiewicz and Musser, 2009).

### Metal ion homeostasis

Metal ion homeostasis in streptococci plays a central role for colonization and invasion. It is involved in enabling adhesins to interact with host surfaces, supporting streptococcal growth in nutrient limiting niches, tolerating oxidative stress, evading host innate immune defenses and producing biofilms (Nobbs et al., 2009). Of special interest in this regard are manganese and iron transporters. Both ions are crucial for bacterial pathogenesis and are known to be highly restricted inside the host. Several investigations propose that the uptake and response to metal ions, such as Mn^2+^ and Fe^2+^, is vital for virulence and physiology of pathogenic streptococci (Kloosterman et al., 2008). Cytoplasmic Mn^2+^ can help in protecting bacteria against oxidative stress, and the induction of Mn^2+^ uptake by H_2_O_2_ has been observed in many bacteria (Horsburgh et al., 2002). Iron represents an essential cofactor for the function of several enzymes. It acts as a catalyst in electron transport processes (Somerville and Proctor, 2009; Diaz-Ochoa et al., 2014) and could indirectly play a role in the anti-oxidative response (Echave et al., 2003).

Connections between acid stress and metal ion homeostasis have been addressed in several studies. Transcriptional analyses of acid tolerance responses in streptococci have reported a remarkable up-regulation of different metal-ion transporters. In *S. pneumoniae* (Martin-Galiano et al., 2005) elevated expression levels of genes belonging to the *psaABC* and putative *fatDCBE* operons were observed which are involved in manganese (Dintilhac et al., 1997; Johnston et al., 2006) and iron transport (Hoskins et al., 2001). Similarly, the expression of *sloR* could be linked to the acid tolerance response in *S. mutans* (Gong et al., 2009) (Idone et al., 2003; Dunning et al., 2008). The *sloR* gene encodes a metal-dependent transcriptional regulator of the *sloABCR* operon, which is responsible for Mn and Fe uptake (Paik et al., 2003). Genes encoding metal ion transporters of nickel, Fe^2+^ and Mn^2+^, as well as genes encoding ABC transporter systems for the import of zinc and molybdenum, were found to be up-regulated in response to acid stress. The authors attributed this to an increased need for essential metals during acid stress. Likewise, for GBS, Santi et al. (2009) reported higher expression levels of several Mn^2+^ and Fe^2+^ transporters upon shifting from pH 7 to pH 5.5. These included members of the *fhuCDBG* operon coding for a siderophore-dependent iron transporter (Clancy et al., 2006), the putative iron-compound ABC transporter operon (Glaser et al., 2002; Tettelin et al., 2005) and members of the *mtsABC* operon (Glaser et al., 2002; Tettelin et al., 2005) which represents of of the Mn^2+^ and Fe^2+^ transporters (Bray et al., 2009) in GBS.

Another metal ion transporter reported by Santi et al. (2009) to be up-regulated in response to acid stress is the *mntH* gene coding for a metal ion NRAMP transporter of GBS. This transporter is predicted to participate in Mn^2+^ and Fe^2+^ homeostasis (Glaser et al., 2002; Tettelin et al., 2005). Interestingly, a recent investigation indicated a direct connection between acid stress and the expression of the NRAMP transporter *mntH* in GBS (Shabayek et al., 2016). MntH was characterized as crucial for GBS growth and survival under low pH conditions and essential for prolonged intracellular survival inside acidic host compartments. In line with these observations, global transcription studies in GBS (Di Palo et al., 2013) reported *mntH* to be regulated by the CovRS system which has been shown to be directly implicated in the GBS response to acid stimuli. Furthermore, previously bacterial NRAMP homologs have been characterized as pH-dependent transporters of divalent metal ions with preference for Mn^2+^ and Fe^2+^ in the following bacterial species *E. coli, S. typhimurium, Mycobacterium tuberculosis* and *B. subtilis* (Agranoff et al., 1999; Kehres et al., 2000; Makui et al., 2000; Que and Helmann, 2000).

### Oxidative and acidic stress

Aerobic conditions are potentially harmful for bacteria due to the formation of highly reactive oxygen species (ROS), such as superoxide anions, hydrogen peroxide and hydroxyl radicals. ROS are toxic leading to irreversible DNA and protein damage (Fridovich, 1978; Imlay and Linn, 1988) and subsequent microbial killing. The primary microbial defense mechanisms to counteract these lethal effects is the release of superoxide dismutases (SODs) (Rolfe et al., 1978). The Mn-dependent SOD, encoded by *sodA* gene, is a major mechanism for ROS detoxification especially in streptococci, which lack a catalase (Poyart et al., 1998). Interestingly, several reports have shown *sodA* to play a significant role in acid resistance and acid-adaptive responses, underlining important overlaps between oxidative and acidic stress. Wilkins et al. (2002) demonstrated SOD of *S. mutans* among the up-regulated proteins in response to low pH. Similarly, SOD induction was also found in *S. oralis* when cells are cultured at pH 5.2 vs. pH 7 (Wilkins et al., 2003). The correlation of SOD expression with acidic conditions does not seem to be restricted to streptococci. A *sodA* mutant of *S. aureus* was shown to be more sensitive to acid stress in comparison to the parental strain (Clements et al., 1999). Moreover, SOD was reported to be crucial for acid tolerance in *Vibrio vulnificus* where *sodA* mutants suffered higher killing rates at pH 5 than at pH 7.5 in comparison to the wild-type strain (Kim et al., 2005).

In GBS with a lack of catalase activity SOD is fundamental in neutralizing oxidative stress (Poyart et al., 1998). Despite a previous investigation reporting SOD to be down-regulated upon exposure to low pH in GBS (Santi et al., 2009), there is evidence for the indirect involvement of the manganese dependent SOD in acid resistance. MntH, belonging to the NRAMP family of Mn transporters has been identified as essential for GBS growth under low pH conditions and in response to ROS (Shabayek et al., 2016). Both *sodA* and *mntH* were found to be induced at low pH in this recent investigation. These results support an interpretation were GBS SOD activity is dependent on MntH activity to provide optimum intracellular levels of manganese, its metal ion cofactor. A similar correlation between Mn homeostasis and SOD activity was reported earlier for *Brucella abortus* (Anderson et al., 2009) and *S. pyogenes* (Janulczyk et al., 2003).

Other strategies used to prevent ROS formation in streptococci include NADH oxidases. There are two genes, *nox-1* encoding a H_2_O_2_-forming NADH oxidase which promotes the reduction of oxygen to H_2_O_2_ whereas *nox-2* encodes a H_2_O-forming NADH oxidase which catalyzes the reduction of oxygen to water without forming harmful ROS intermediates (Higuchi et al., 1993, 1999). Both genes were described in *S. mutans* (Higuchi et al., 1993, 1999), however, only *nox-2* gene was investigated in most other streptococci including GBS (Gibson et al., 2000; Yu et al., 2001; Yamamoto et al., 2006; Ge et al., 2016). Yamamoto et al. (2006), characterized Nox-2 as the main NADH oxidase that is required for aerobic growth and oxidative stress in conventional culture media in GBS. Moreover, addition of antioxidants did not alleviate aerobic growth defect in the GBS *nox-2* mutant, however, it could be restored by the addition of exogenous unsaturated fatty acids. Previous reports in streptococci have related membrane fatty acid composition with acid tolerance (Kajfasz and Quivey, 2011; Quivey et al., 2016). Even more, concurrent acid and oxidative stresses have been shown to be associated with elevated unsaturated fatty acid abundance (Derr et al., 2012). Taken together, these data suggest that the aerobic growth defect of the GBS *nox-2* mutant is due to the impaired ability to cope with the simultaneous acid stress induced by lactic acid, a growth by-product, as confirmed by the altered membrane fatty acid composition. Aerobic growth in GBS is fermentive and results mainly in acid production in the absence of heme and quinone (Yamamoto et al., 2005). In agreement, the *nox-2* mutant of *S. sanguinis* was shown to be more sensitive to oxidative stress and acid stress (Ge et al., 2016). Furthermore, cultures of *S. mutans* UA159 exposed to concurrent acid and oxidative stresses demonstrated elevated levels of *nox-2* transcription than under either stress alone (Baker et al., 2014, 2015b). Unfortunately, studies characterizing the direct involvement of Nox-2 in GBS acid stress are not available.

The transcription of *nox* have been shown to be modulated by *rex* which encodes the redox-sensing regulator Rex. This is a NAD^+^/NADH sensing transcription factor that is active when bound to NAD^+^ and inactive when bound to NADH. It senses the NAD^+^/NADH ratio in the cell and controls its regulon accordingly (Bitoun and Wen, 2016). The Rex regulator is ubiquitously conserved across Gram-positive bacteria (Ravcheev et al., 2012; Bitoun and Wen, 2016). It has been characterized in *S. mutans* (Bitoun et al., 2011), *E. faecalis* (Vesic and Kristich, 2013), and recently in *S. pneumoniae* (Luong et al., 2015). Although genome sequencing deduced Rex to be present in GBS (Bitoun and Wen, 2016), it has not yet been characterized.

Cytochrome bd quinol oxidase (CydABCD) is another system that is involved in protection against oxidative stress. This system has been shown to be essential for the respiratory metabolism and establishing a full respiration chain in GBS when the surrounding environment supplies heme and quinone. Aeration alone had a slight effect on aerobic growth in comparison to static conditions in GBS as indicated by Yamamoto et al. (2005). Interestingly, the authors observed that aerobic growth in GBS was mainly fermentive resulting in lower pH culture values in the absence of heme and quinone. They also observed that *cydA* expression was induced late in growth. In concordance, Santi et al. (2009) reported all the four subunits of the Cytochrome bd quinol oxidase system to be upregulated in response to acid stress.

Oxidative stress tolerance in streptococci is also driven by alkayl hydroperoxidase (AhpCF) and thiol peroxidase (Tpx) (Lemos et al., 2011; Papadimitriou et al., 2016). In *S. mutans*, peroxidase activity is mainly achieved through the AhpC-AhpF complex where AhpF (or Nox-1) acts as a dehydrogenase that reduces NADH and delivers electrons to AhpC. In addition, AhpC converts peroxidase into water or alchohol (Lemos et al., 2011). Transcriptional profile analysis displayed the upregulation of *ahpCF* upon transition from steady-state growth at pH 7 to steady-state growth at pH 5 in *S. mutans* (Baker et al., 2015a). Surprisingly, AhpC was characterized as a heme-binding protein that is required for complete respiration activity in GBS. It was demonstrated to be involved in managing intracellular heme (Lechardeur et al., 2010). A direct contribution of AhpC in GBS acid tolerance has not been reported. Thiol peroxidase (Tpx) is a member of the peroxiredoxin family of antioxidant enzymes, which collect electrons from a reducing system containing thioredoxin and thioredoxine reductase (La Carbona et al., 2007). Genome-wide transcription analysis in GBS demonstrated *tpx* gene to be induced in response to acid stress (Santi et al., 2009). This is consistent with the observation of significantly diminished intracellular survival of a *E. faecalis tpx* mutant inside mouse peritoneal macrophages in comparison to mutants for the NADH peroxidase *npr* and the alkayl hydroperoxidase *ahpCF* genes (La Carbona et al., 2007). However, Tpx has not been characterized in GBS and remains to be investigated.

## Conclusion

In GBS a wealth of data has accumulated from genome sequencing projects, genome-wide transcription analysis and proteome mapping resulting in the large-scale identification of genes and proteins that are induced in response to low-pH environments. However, care must be taken when interpreting these results, to differentiate between genes and proteins with a direct contribution in the pH stress response and more unspecific effects. To validate these results, more conventional techniques need to be employed, such as the construction of mutants and addressing the consequences of these mutations on an individual basis. Unfortunately, functional studies to investigate GBS acid responses are still rare. Nonetheless, this review shows that genomic and proteomics-based approaches have identified many acid-inducible genes, such as the ADI system genes, F1F0-ATPase genes, genes of the glycine betaine system, genes encoding Clp chaperones, metal-ion transporters especially for Mn^2+^ and Fe^2+^, the Mn-dependent SOD, cytochrome bd quinol oxidase and thiol peroxidase. These genes can be targeted in further mutagenesis studies to assess and confirm their physiological significance for GBS in coping with acid stress. As discussed above, a successful scenario was recently shown when proofing the direct contribution of the *mntH* NRAMP transporter in GBS acid stress that was first mentioned in an earlier genome-wide transcription study.

## Author contributions

BS and SS both designed and wrote the manuscript.

### Conflict of interest statement

The authors declare that the research was conducted in the absence of any commercial or financial relationships that could be construed as a potential conflict of interest.
